# CCL5 mediates target‐kinase independent resistance to FLT3 inhibitors in FLT3‐ITD‐positive AML

**DOI:** 10.1002/1878-0261.12640

**Published:** 2020-02-13

**Authors:** Silvia Waldeck, Michael Rassner, Philip Keye, Marie Follo, Dieter Herchenbach, Cornelia Endres, Anne Charlet, Geoffroy Andrieux, Ulrich Salzer, Melanie Boerries, Justus Duyster, Nikolas von Bubnoff

**Affiliations:** ^1^ Department of Medicine I (Hematology, Oncology and Stem Cell Transplantation) Medical Center Faculty of Medicine University of Freiburg Germany; ^2^ Faculty of Biology University of Freiburg Germany; ^3^ German Cancer Consortium (DKTK) partner site Freiburg and German Cancer Research Center (DKFZ) Heidelberg Germany; ^4^ Department of Ophthalmology Medical Center Faculty of Medicine University of Freiburg Germany; ^5^ Institute of Medical Bioinformatics and Systems Medicine Medical Center Faculty of Medicine University of Freiburg Germany; ^6^ Center for Chronic Immunodeficiency Medical Center Faculty of Medicine University of Freiburg Germany; ^7^ Department of Rheumatology and Clinical Immunology Medical Center Faculty of Medicine University of Freiburg Germany; ^8^ Department of Hematology and Oncology Medical Center University of Schleswig Holstein Lübeck Germany

**Keywords:** CCL5/RANTES, FLT3‐ITD, PKC412, TKI resistance

## Abstract

FLT3‐ITD tyrosine kinase inhibitors (TKI) show limited clinical activity in acute myeloid leukemia (AML) due to emerging resistance. TKI resistance is mediated by secondary FLT3‐ITD mutations only in a minority of cases. We hypothesize that the cytokine CCL5 protects AML cells from TKI‐mediated cell death and contributes to treatment resistance. We generated PKC412‐ and sorafenib‐resistant MOLM‐13 cell lines as an *in vitro* model to study TKI resistance in AML. Increased CCL5 levels were detected in supernatants from PKC412‐resistant cell lines compared to TKI‐sensitive cells. Moreover, CCL5 treatment of TKI‐sensitive cells induced resistance to PKC412. In resistant cell lines with high CCL5 release, we observed a significant downregulation of the CCL5‐receptor CCR5 and CXCR4. In these cell lines, TKI resistance could be partly overcome by addition of the CXCR4‐receptor antagonist plerixafor. Microarray and intracellular flow cytometry analyses revealed increased p‐Akt or p‐Stat5 levels in PKC412‐resistant cell lines releasing high amounts of CCL5. Treatment with the CXCR4 antagonist plerixafor, αCCL5, or CCR5‐targeting siRNA led to a decrease of p‐Akt‐positive cells. Transient transfection of sensitive MOLM‐13 cells with a CCL5‐encoding vector mediated resistance against PKC412 and led to an increase in p‐Akt‐positive and p‐Stat5‐positive cells. Isolated AML blasts from patients treated with PKC412 revealed that CCL5 transcript levels increase significantly at relapse. Taken together, our findings indicate that CCL5 mediates resistance to FLT3‐TKIs in FLT3‐ITD‐mutated AML and could possibly serve as a biomarker to predict drug resistance.

## Graphical abstract

Increased signaling of the CCL5‐CCR5 pathway can protect FLT3‐ITD^+^ AML cells from cell death mediated by tyrosine kinase inhibitors like midostaurin. Thereby, either increased CCL5 release or upregulation of the CCR5/CXCR4 receptors induces enhanced levels of p‐AKT, p‐STAT5, and Bcl‐2, possibly mediating enhanced survival. Therefore, CCL5 could pave the way for new treatment strategies in FLT3‐ITD^+^ AML patients at relapse.

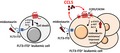

**Abbreviations**
AML: acute myeloic leukemia
FLT3: fms‐like tyrosine kinase 3
ITD: internal tandem duplication
TKI: tyrosine kinase inhibitor

## 1 Introduction

Activating mutations of fms‐like tyrosine kinase 3 (FLT3) occur in approximately 35% of AML patients (Thiede *et al.*, 2002). Most of these mutations are internal tandem duplications (ITD) in the juxtamembraneous domain and are associated with poor prognosis (Barry *et al.*, 2007). Small molecule FLT3 tyrosine kinase inhibitors (TKI) such as PKC412 (midostaurin) and sorafenib are currently tested in clinical trials. The addition of PKC412 to standard chemotherapy significantly improved overall survival in untreated and treated AML patients with FLT3 mutations and decreased relapse risk after allogenic bone marrow transplantation (Stone *et al.*, 2017). PKC412 was just recently approved in the United States and Europe for the treatment of FLT3‐mutated AML patients. However, in FLT3‐mutated patients treated with single‐agent FLT3‐TKI complete remissions are rare, responses are short‐lived, and the majority of patients display primary or secondary resistance (Fischer *et al.*, 2010; Knapper *et al.*, 2006). Acquired resistance to FLT3‐TKIs was related to secondary FLT3 mutations in only a small number of cases (von Bubnoff *et al.*, 2010; Heidel *et al.*, 2006; Smith *et al.*, 2012). For this reason, additional mechanisms such as protective serum soluble factors might be the cause of the drug resistance.

In several tumor entities, cytokines were found to contribute to drug resistance. In AML, KIT inhibition by imatinib and nilotinib was shown to be overcome in the presence of the cytokine G‐CSF (Gordon *et al.*, 2014). The cytokine C‐C motif chemokine 5 (CCL5, previously known as RANTES) is physiologically a regulator of immune cell migration and was identified as constituting a distinct chemokine release cluster in AML (Bruserud *et al.*, 2007) and as an important drug resistance mediator in several tumor entities: In breast, ovarian, and prostate cancer, CCL5 rendered tumor cells resistant to tamoxifen, saracatinib, cisplatin, or taxanes, respectively (Fang *et al.*, 2018; Kato *et al.*, 2013; Pasquier *et al.*, 2018; Xiang *et al.*, 2019; Yi *et al.*, 2013; Zhou *et al.*, 2016). In addition, CCL5 was found to contribute to tumor progression and metastasis (Azenshtein *et al.*, 2002; Velasco‐Velázquez *et al.*, 2012). Importantly, CCL5 was identified in several cancers as a useful biomarker for predicting treatment response and disease progression (Suenaga *et al.*, 2016).

In the present study, we examined the role of CCL5 in FLT3‐TKI‐resistant AML. Our data suggest that CCL5 release is one factor that contributes to FLT3‐TKI resistance, possibly via Stat5 or PI3K/Akt and Bcl‐2 signaling *in vitro* and is upregulated *ex vivo* in blasts from FLT3 mutated AML patients preceding failure to FLT3‐TKI therapy.

## 2 Materials and methods

### 2.1 Cell lines

To investigate the underlying mechanisms that induce TKI resistance in AML, TKI‐resistant cell lines were established using a cell‐based resistance screen as described previously (von Bubnoff *et al.*, 2005). Briefly, MOLM‐13 cells were plated into 96‐well plates at a density of 4 × 10^5^ cells/well in liquid RPMI medium and were cultured for 28 days with a FLT3 inhibitor concentration of 100 nm PKC412 or 40 nm sorafenib responding to IC_90_ determined in sensitive MOLM‐13 cells (data not shown). When visible resistant cell clones grew up, they were aspirated using a pipette, expanded, and maintained in the presence of 100 nm PKC412 or 40 nm sorafenib. MOLM‐13 cells, which are heterozygous for FLT3‐ITD, were kindly provided by M. Luebbert (Freiburg, Germany). Cell lines were maintained at 37°C with 5 % CO_2_ in RPMI (Gibco/Thermo Fisher Scientific, Waltham, MA, USA) supplemented with 20 % FBS (Biochrom, Berlin, Germany). PKC412 was a kind gift from Novartis Pharma AG, Basel, Switzerland. Sorafenib was purchased from American Chemicals Custom Corporation, San Diego, CA USA. Each compound was dissolved in dimethyl sulfoxide (DMSO) to give a 10 mm stock solution and stored at −20°C.

### 2.2 Cytokine array

The cytokine expression profile in the supernatant of MOLM‐13 PKC412 and sorafenib‐resistant cell lines was analyzed using Human Cytokine Antibody Arrays 3.0” (Affymetrix, Santa Clara, CA, USA) according to the manufacturer’s protocol. These arrays comprise detection of CCL5, CCL3, CCL4, CTLA, Apol/Fas, Eotaxin, GM‐CSF, EGF, IP‐10, Leptin, MIP4, MIP‐5, MMP3, TGF‐β, IFN‐γ, TNF‐α, TNFRI, TNFRII, ICAM‐1, VCAM‐1, VEGF, IL‐1α, IL‐1β, IL‐1ra, IL‐2, IL‐3, IL‐4, IL‐5, IL‐6, IL‐6R, IL‐7, IL‐8, IL‐10, IL‐12(p40), IL‐15, IL‐17. For analysis, cell culture supernatant was collected 36 h after adding fresh culture medium. Supernatant from nonresistant MOLM‐13 cells as well as RPMI culture medium with 20 % FBS (Gibco/Thermo Scientific) served as a negative control.

### 2.3 ELISA

CCL5 and SDF1‐α secretion of resistant MOLM‐13 cell lines was analyzed using ABTS Elisa KITs (PeproTech, Rocky Hill, CT, USA) according to the manufacturer’s instructions. Cell culture supernatant was analyzed 36 h after supply with fresh culture medium.

### 2.4 Flow cytometry

For apoptosis staining, 1 × 10^6^ cells of each sample were stained with anti‐Annexin‐V (BD Biosciences, San Jose, CA, USA) and 7‐AAD (eBioscience, San Diego, CA, USA). For staining of CCR5 and CXCR4, 1 × 10^6^ cells per sample were stained with anti‐human CCR5 PE‐conjugated antibody (R & D, Minneapolis, MN, USA) or PE anti‐human CD184 (BD Pharmingen, San Jose, CA, USA). Intracellular p‐Akt, p‐Stat5, and Bcl‐2 staining was performed with 1 × 10^6^ cells, which had been starved overnight, according to the manufacturer’s protocol. Briefly, cells were fixed with Cytofix Buffer (BD Biosciences) at 37°C for 15 min. Afterward, cells were permeabilized for 30 min on ice using Perm Buffer III (BD Biosciences, San Jose, USA) followed by two washing steps and were stained with Alexa 647 anti‐p‐Akt (pS473) (BD Biosciences), Alexa 647 anti‐p‐Stat5 (pY694) (BD, Biosciences) for 1 h or with PE anti‐Bcl‐2 (BD Biosciences) for 30 min. All samples were analyzed on a Fortessa cytometer (BD Biosciences), and data analysis was performed using flowjo V10 software (FlowJo LLC, Ashland, OR, USA).

### 2.5 Microarray

Microarrays for mRNA expression analysis were purchased from Affymetrix, and the analysis was conducted according to manufacturer’s protocol. Briefly, RNA intensity was normalized using the Single‐Channel Array Normalization (SCAN) method (Piccolo *et al.*, 2012). Differential analysis was performed with a linear model‐based approach (limma), and genes with adjusted *P*‐value below 0.05 were considered significant (Ritchie *et al.*, 2015).

### 2.6 MTS assay

To determine the IC_50_ for PKC412‐sensitive and PKC412‐resistant cell lines, 1 × 10^4^ cells/well were cultured in 96‐well plates with increasing concentrations of PKC412 (5 to 1000 nm). Also, cell viability and proliferation at 5 to 1000 nm PKC412 with or without 50 ng/ml CCL5 (PeproTech) and with or without 500 nm TAK‐779 (Sigma‐Aldrich, St. Louis, MO, USA) were measured. After 48 h, 20 μL MTS solution (Promega, Madison, WI, USA) was added to each well and the cells were incubated for an additional 2 h. Absorbance at 490 nm was measured with a 96‐well plate reader (Tecan, Männedorf, Switzerland) in accordance with the manufacturer’s instructions. The relative viability was calculated by dividing the measured value of each well by the measurement of the untreated control. The relative IC_50_ was calculated by nonlinear regression analysis with graphpad version 4.0 software (GraphPad Software).

### 2.7 Chemotaxis studies

The chemotactic behavior of MOLM‐13 cells was assessed with HTS Transwell plates (Corning, New York, USA) according to the manufacturer’s instructions. Cell culture supernatant from PKC412‐sensitive or PKC412‐resistant MOLM‐13 cell lines was added to 96‐ or 24‐well plates. TKI‐sensitive MOLM‐13 cells were then added to the upper chamber at a density of 3 × 10^5^ cells in 100 μL RPMI for a 24‐well plate or 1 × 10^5^ cells in 50 μL RPMI for a 96‐well plate. After 2 h of incubation at 37°C, the number of cells in the lower chamber was counted with a flow cytometer (setting: FSC SSC, dead cells were excluded). For each condition, the chemotaxis index was calculated as the ratio between the measured number of cells in three treated wells and the measured number of cells in three control wells filled with RPMI.

**Table 1:** IC_50_ in PKC412‐ and sorafenib‐sensitive and PKC412‐ and sorafenib‐resistant cell lines. 1 × 10^4^ cells were cultured with increasing concentration of PKC412 (5–1000 nm). Cell viability was measured after addition of MTS. The respective IC_50_ was calculated by nonlinear regression analysis (log inhibitor vs. response). Mean IC_50_ (*n* = 6; 2 experiments) with standard error (SE) as LogIC_50_ is depicted

PKC412			Sorafenib		
Cell line	IC_50_ in nmol/l	SEM as LogIC_50_	Cell line	IC_50_ in nmol/l	SEM as LogIC_50_
MOLM13	32.17	0.05	MOLM13	4.66	0.05
PKC C5	105.0	0.03	Sora C4	267	0.04
PKC E9	136.2	0.03	Sora D3	291	0.12
PKC F6	147.0	0.07	Sora D11	63.9	0.16
PKC F9	156.1	0.02	Sora E7	264	0.15
PKC G7	170.5	0.08	Sora G8	68.1	0.10

### 2.8 Constructs and *in vitro* transfection

Transient transfections in MOLM‐13 cells were performed by using Lipofectamine 2000 (Life Technologies, Carlsbad, CA, USA) for a CCL5 encoding plasmid or Lipofectamine RNAiMax (Life Technologies) for siRNA, respectively. A CCL5‐encoding pcDNA 3.1/Zeo(‐) plasmid was purchased from GenScript, Piscataway, NJ, USA, and an amount of 10 µg was used to transfect 5 × 10^5^ MOLM‐13 cells. siRNA targeting CCR5 was designed via webtool (Thermo Fisher) and ordered from Thermo Fisher.

siRNA 1:

forward 5′‐GCUUCUUCUCUGGAAUCUUTT‐3′

reverse 5′‐AAGAUUCCAGAGAAGAAGCTT‐3′

siRNA 2:

forward 5′‐CCAUACAGUCAGUAUCAAUTT‐3′

reverse 5′‐AUUGAUACUGACUGUAUGGTT‐3′

A final concentration of 20 nm siRNA (optimal concentration determined in dilution experiments, data not shown) was used to knock down CCR5 expression in PKC412‐resistant MOLM‐13 cells.

### 2.9 Patient samples

This study was conducted in accordance with the Declaration of Helsinki after approval by the local institutional review board (ethics commission of the University of Freiburg, ethical approval nr. 528/16), and written and informed consent of the patients had been obtained. Bone marrow or peripheral blood mononuclear cells from 16 AML patients (age: 35–83 years) were collected at initial diagnosis and at either relapse or from patients that did not achieve complete hematological remission after they had been treated with chemotherapy and/or FLT3‐targeted treatment previously. The mononuclear cells were isolated using a Ficoll density gradient. Cells were stored in liquid nitrogen until further use.

### 2.10 Plerixafor treatment

Plerixafor was purchased from SellCheck (Selleckchem, Munich, Germany). Cells were incubated simultaneously with 100 nm PKC412 and different concentrations of plerixafor (250 nm, 1 μM) for 36 h when analyzing apoptosis. During the incubation, plerixafor was added every 24 h. For analysis of p‐Akt via flow cytometry, plerixafor was used at a concentration of 1 µm and added at different time points before analysis.

### 2.11 RNA isolation and cDNA synthesis

Total RNA was isolated with the RNeasy Mini Kit (Qiagen, Hilden, Germany) for AML cell lines or with the AllPrep DNA/RNA Mini Kit (Qiagen, Hilden, Germany) for human patient samples, respectively. 500 ng of RNA was transcribed into cDNA with the Maxima First Strand cDNA synthesis Kit that contains random hexamer primers (Thermo Scientific) according to the manufacturer’s protocol.

### 2.12 Sanger sequencing

For Sanger sequencing of the human FLT3 kinase domain exons 11 to 24, a 1600‐bp region was amplified using the following primers:

forward 5`‐GTCCTGTTTCTCGGATGGATACC‐CATTAC‐3`;

reverse 5`‐CTACGAATCTTCGACCTGAGCCTGCGGAGAGA‐3`.

The resulting PCR product was purified with Exo‐Sap‐it (Affymetrix, Santa Clara, USA) and sequenced with the following primers diluted to 5 pmol/μL:

huFLT3TK1 forward 5`‐GCAACAATTGGTGTTTGTCTCCTC‐3`;

huFLT3TK1rev 5`‐GGTCTCTGTGAAC‐ACACGACTTAAAT‐3`;

huFLT3TK2for 5`‐CAGATACACCCGGACTCGGATCAA‐3`;

huFLT3TK2rev 5`‐GTGAGGACATTCCGAAACACGGCCAT‐3`.

### 2.13 Quantitative real‐time PCR

For quantitative PCR of CCL5, CCR1, CCR3, CCR5, GAPDH, and ABL, primers for CCR1, CCR3, CCR5 were designed according to Okita *et al.* (2005) and for GPR75 according to Sauer *et al.* (2001). Five microliters of cDNA was combined with 16 µL Master Mix consisting of 12.5 µL Lightcycler 480 Master Mix (Roche, Basel, Switzerland), 2.5 µL LC Green (BioFire Defense, Murray, USA), and 1 µL distilled water per sample. Two microliters of reverse and forward primers (2.5 pmol/µL) were added. The RT‐/qPCRs were run in duplicates on a Rotor‐Gene Q (Qiagen, Hilden, Germany). In general, cycling conditions were as follows: 1 cycle 95°C for 10 min, followed by 50 cycles at 95°C for 15 sec and 60°C for 1 min. Depending on the target, slightly adjustments were made to the cycling conditions. The relative expression was calculated by the delta–delta‐Ct method. ABL and GAPDH were used as reference genes.

### 2.14 Statistics

Statistical analysis was performed using graphpad version 4.0 software (GraphPad Software). Data are presented as arithmetic means with SD. A two‐sided Student’s t‐test was used to analyze quantitative variables between two groups with *P* < 0.05 indicating a statistically significant difference.

## 3 Results

### 3.1 PKC412‐resistant MOLM‐13 AML cells secrete CCL5

**Figure 1:**
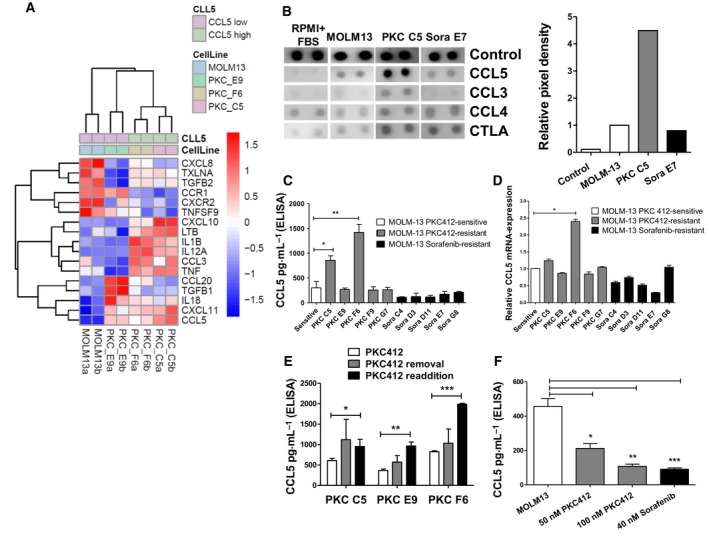
High levels of CCL5 in the supernatant of PKC412‐resistant MOLM‐13 cells and increased expression of mRNA in PKC412‐resistant MOLM‐13 cell lines (A) Microarray results for cytokine/chemokine expression with RNA from three different PKC412 and MOLM‐13 sensitive cells**.** Color code represents the row‐wise *z*‐score intensity. *N* = 2. (B) Cytokine array of supernatant from one PKC412‐ (MOLM‐13 PKC C5) and one sorafenib‐resistant MOLM‐13 cell line (MOLM‐13 Sora E7). Medium supplemented with 20 % FBS and supernatant from sensitive MOLM‐13 cells served as controls. Supernatants were subjected to analysis 36 h after the addition of fresh culture medium. (C) CCL5 ELISA with supernatant from five PKC412‐ and five sorafenib‐resistant cell lines. Supernatants were subjected to analysis 36 h after the addition of fresh culture medium. (D) Quantitative reverse‐transcriptase PCR for CCL5 in TKI‐sensitive, PKC412‐resistant, and sorafenib‐resistant cell lines. Differences between data were compared by ANOVA. (E) CCL5 ELISA with cell supernatant from three different PKC412‐resistant cell lines after removal of and re‐exposure to PKC412. The supernatants were analyzed 36 h after removal of and 36 h after re‐exposure to PKC412. *n* = 3 (F) CCL5 ELISA with supernatant from sensitive MOLM‐13 cells that were cultured in the presence of PKC412 at 50 or 100 nm or 40 nm sorafenib. Cell culture supernatants were analyzed 36 h after the addition of fresh culture medium containing the FLT3 inhibitors. Significant differences are marked with (*): *P < 0.05, **P < 0.01, ***P < 0.001. If not otherwise stated: Student’s *t*‐test. Error bars represent SD. *N* = 3 for (C–F).

The IC_50_ for PKC412 and sorafenib in MOLM13 cell lines was determined in MTS assays by nonlinear regression analysis (Table 1). Five PKC412‐ and five sorafenib‐resistant MOLM‐13 AML cell lines were generated by selection and expansion in the presence of 100 nm PKC412 or 40 nm sorafenib—concentrations of both TKIs corresponding to IC_90_ values for growth suppression of TKI‐sensitive MOLM‐13 cells. Resulting cell lines were PKC C5, E9, F6, F9, G7 and the sorafenib‐resistant cell lines Sora C4, D3, D11, E7, and G8. Higher IC_50_ values of these cell lines were observed in MTS assays (Table 1). By means of Sanger sequencing, we excluded secondary resistance mutations in the FLT3 kinase domain (data not shown). Flow cytometry analysis for viable cells confirmed that these cell lines were resistant to PKC412 up to 100 nm (Fig. S1). mRNA from three different PKC412‐resistant cell lines and the sensitive MOLM‐13 cell line, respectively, was examined in microarray studies: CCL5 expression was found to be upregulated in all three PKC412‐resistant cell lines compared to sensitive MOLM‐13 cells (Fig. 1A). To verify this at the protein level, supernatant from one PKC412‐ and one sorafenib‐resistant MOLM‐13 cell line was subjected to cytokine array analysis. In contrast to sensitive MOLM‐13 cells and the sorafenib‐resistant cell line, an elevated level of CCL5, but not CCL3, CCL4, or CTLA was detected in the supernatant of the PKC412‐resistant cell line MOLM‐13 PKC C5 (Fig. 1B). We did not detect any other cytokine induced in the supernatants of TKI‐resistant cells. The array results were confirmed by CCL5 ELISA: In two out of five PKC412‐resistant cell lines (PKC C5 and F6), we detected significantly higher CCL5 protein levels in the supernatant compared to sensitive MOLM‐13 or sorafenib‐resistant MOLM‐13 cell lines (Fig. 1C). Additionally, mRNA expression of these cell lines demonstrated increased CCL5 transcription in the cell line PKC F6 (Fig. 1D). To exclude induction of CCL5 secretion by PKC412‐treatment, we withdrew PKC412 from three PKC412‐resistant cell lines for 36 h. We then measured CCL5 secretion by ELISA and did not detect a decrease in the CCL5 release after removal of PKC412 (Fig. 1E). However, re‐exposure to PKC412 induced high CCL5 secretion in two cases. In contrast, in PKC412‐sensitive MOLM‐13 cells, the addition of 50 or 100 nm PKC412 as well as 40 nm sorafenib did not induce CCL5 release after 36 h—a time point at which a high proportion of cells was still viable (Fig. 1F).

### 3.2 CCL5 treatment renders sensitive MOLM‐13 cells resistant to PKC412

**Figure 2:**
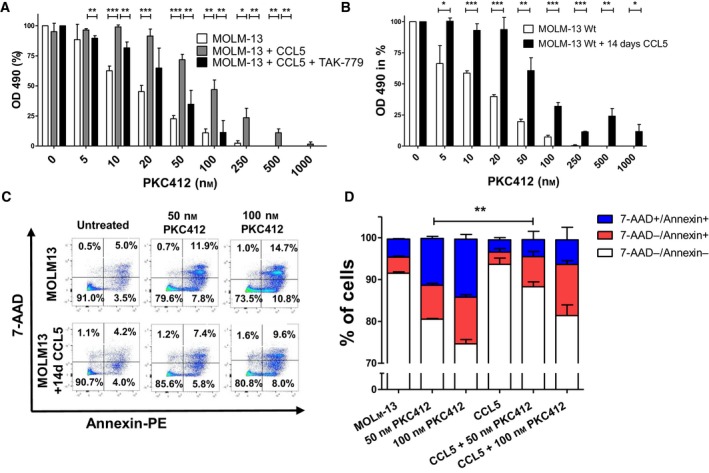
CCL5 mediates partial protection from PKC412 induced growth suppression and apoptosis in FLT3‐ITD‐positive MOLM‐13 cells (A) PKC412‐sensitive MOLM‐13 cells were cultured in the presence of 50 ng/mL CCL5 ± 500 nm TAK‐779 and simultaneously treated with PKC412 at the indicated concentrations. MOLM‐13 cells cultured without CCL5 and treated with PKC412 served as a control. After 48 h, proliferation was measured in MTS analysis. Three independent experiments were performed in triplicates. (B) TKI‐sensitive MOLM‐13 cells were cultured in the presence of CCL5 at 50 ng/mL for 14 days followed by treatment with PKC412 at the indicated concentrations. Proliferation was measured using MTS assay 48 h after addition of PKC412. (C) Sensitive MOLM‐13 cells were cultured in the presence of CCL5 at 50 ng/mL for 14 days or left untreated and after 14 days were exposed to PKC412 (50, 100 nm) for 36 h. Flow cytometric analysis of vital (Annexin‐PE‐negative, 7‐AAD‐negative) and apoptotic cells (Annexin‐PE‐positive, 7‐AAD‐positive) was performed. (D) Same experiments as in C, depicted as bar chart. Annexin‐PE^+^/7‐AAD^+^ cells labeled as total apoptotic cells, single Annexin‐PE^+^ cells labeled as early apoptotic cells, single 7‐AAD^+^ cells labeled as late apoptotic cells. Significant differences are marked with (*): *P < 0.05, **P < 0.01, ***P < 0.001. Student’s t‐test. Error bars represent SD. *N* = 3 for (A,B and D).

Since two PKC412‐resistant cell lines released high levels of CCL5, we examined the protective effect of CCL5 against PKC412. For this purpose, sensitive MOLM‐13 cells were coexposed to PKC412 and CCL5. After two days, viability was measured via MTS assay. Compared to untreated cells, those treated with CCL5 showed a significantly higher viability (*P* < 0.001 for 50 nm and *P* < 0.01 for 100nm) (Fig. 2A). This effect could be partly abrogated by the CCR5‐receptor antagonist TAK‐779 (Fig. 2A). In addition, the role of long‐term treatment with CCL5 was analyzed. After two weeks of daily CCL5 treatment (50 ng/mL), treated cells again exhibited a significantly higher viability upon exposure to PKC412 compared to MOLM‐13 cells not exposed to CCL5 beforehand (Fig. 2B). Interestingly, sensitive MOLM‐13 cells pretreated with CCL5 for two weeks displayed a significantly lower rate of Annexin^+^/7‐AAD^‐^ and Annexin^+^/7‐AAD^+^ apoptotic cells 36 h after the addition of PKC412 at 50 nm compared to cells not pretreated with CCL5 (*P* < 0.01) (Fig. 2C,D). A trend toward a lower apoptotic rate after PKC412‐treatment was also detected at concentrations of 100 nm PKC412 (Fig. 2C,D).

### 3.3 High CCL5 secretion leads to significantly reduced CCR5‐ and CXCR4‐receptor expression and chemoattraction to PKC412‐sensitive cells

**Figure 3:**
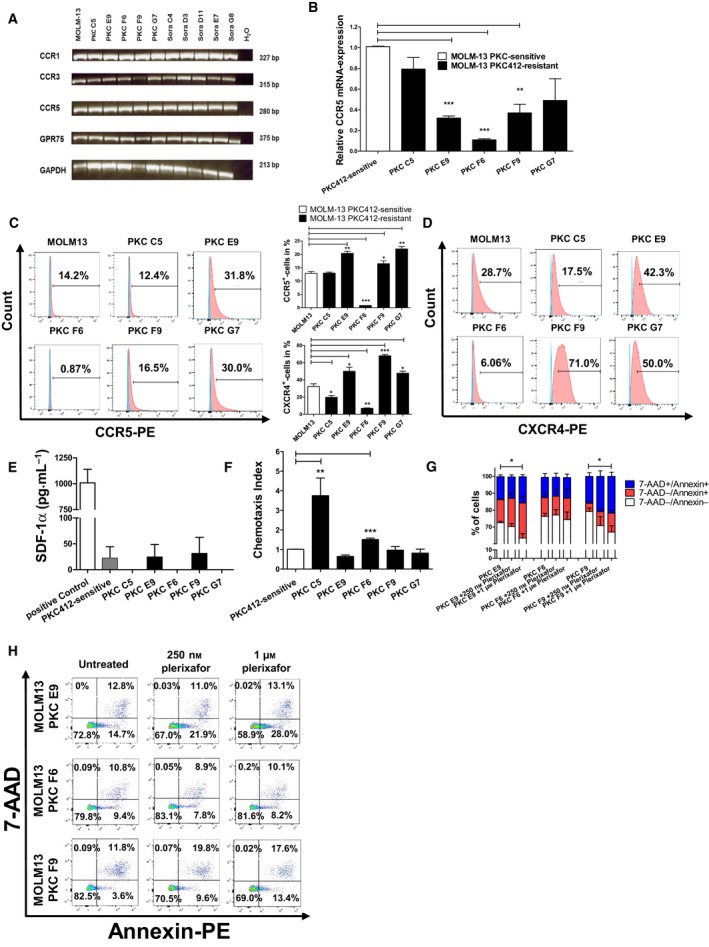
Analysis of CCR1, CCR3, CCR5, and GPR75 expression on sensitive and resistant MOLM‐13 cells and plerixafor treatment (A) Results of reverse‐transcriptase PCR for CCR1, CCR3, CCR5, and GPR75 for mRNA/cDNA isolated from PKC412‐sensitive and PKC412‐resistant MOLM‐13 cells. PCR products were separated each on one gel (merged data). (B) Quantitative real‐time PCR for the main‐CCL5 receptor CCR5 for mRNA/cDNA isolated from sensitive and PKC412‐resistant MOLM‐13 cell lines. (C) Flow cytometric analysis for CCR5 surface expression on PKC412‐sensitive and PKC412‐resistant MOLM‐13 cell lines was conducted with a PE‐coupled antibody against CCR5. Unstained control cells that were used as control are shown in blue and PE‐positive cells are depicted in red. (D) Flow cytometric analysis for CXCR4 on sensitive and PKC412‐ and sorafenib‐resistant MOLM‐13 cell lines was performed with a PE‐coupled antibody against CXCR4. Unstained control cells that were used as controls are shown in blue and PE‐positive cells are depicted in red. (C) + (D) middle: Statistical analyses of flow cytometric CCR5 and CXCR4 stainings. (E) SDF‐1α‐ELISA. Cell culture supernatants from sensitive and PKC412‐resistant MOLM‐13 cells were analyzed 36 h after the addition of fresh culture medium. (F) Chemotaxis of sensitive MOLM‐13 cells against supernatants of PKC412‐resistant MOLM‐13 cells. Cell culture supernatants were collected 36 h after addition of fresh culture medium and added to the lower panel of a chemotaxis chamber. Then, PKC412‐sensitive Molm‐13 cells were added for 2 h to the upper chamber and chemotaxis was measured via flow cytometry. (G) + (H): Three PKC412‐resistant cell lines (PKC E9, F6 and F9) were cultured for 36 h with PKC412 plus either 250 nm or 1 μm plerixafor. Then, the cells were analyzed for vital (Annexin‐PE^‐^, 7‐AAD) and apoptotic cells (Annexin‐PE^+^, 7‐AAD^+^) via flow cytometry. (G) Same experiments as in (H), depicted as bar chart. Significant differences are marked with (*): *P < 0.05, **P < 0.01, ***P < 0.001. Student’s t‐test. Error bars represent SD. *N* = 3 for (B–G).

**Figure 4:**
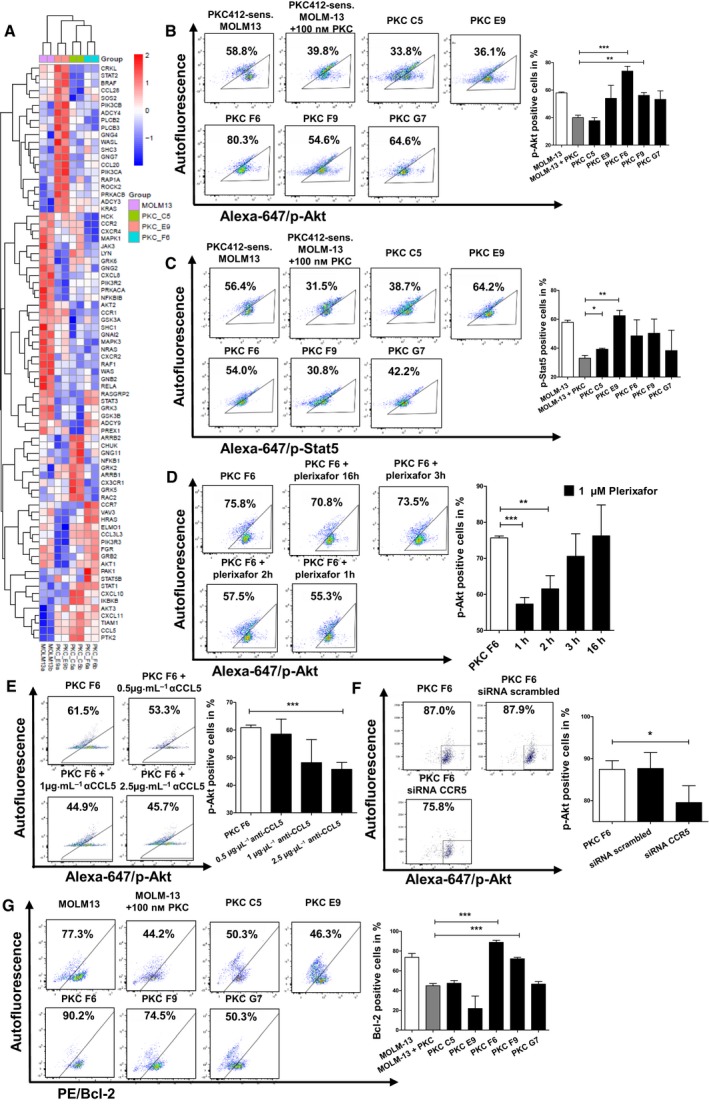
Microarray analysis for differences in mRNA expression between PKC412‐sensitive and PKC412‐resistant cell lines and analysis of p‐Akt, p‐Stat5, and Bcl2 upon plerixafor treatment, anti‐CCL5 inhibitory antibody, or siRNA (A) Selected genes are regulated in at least one of the three PKC412‐resistant cell lines against PKC412‐sensitive MOLM‐13 (adjusted *P*‐value < 0.05) and belong to the KEGG chemokine pathway. Color code represents the row‐wise z‐score intensity. *N* = 2 (B) + (C) PKC412‐sensitive and PKC412‐resistant MOLM13 cells were serum‐starved for 12‐h and incubated without or with 100 nm PKC412. After incubation, cells were analyzed via intracellular flow cytometric analysis for (B) p‐Akt or (C) p‐Stat5**.** (D) The PKC412‐resistant cell line PKC F6 was serum‐starved for 12‐h, while still under cultivation of 100 nm PKC412. Then, plerixafor (1 µm) was added for 1, 2, 3, or 16 h followed by flow cytometric analysis of intracellular p‐Akt levels. (E) The PKC412‐resistant cell line PKC F6 was serum‐starved for 12 h, while still under cultivation of 100 nm PKC412. Then, αCCL5 at the indicated concentrations was added for 1 h followed by flow cytometric analysis of intracellular p‐Akt levels. (F) PKC412‐resistant cells were serum‐starved for 16 h and transfected with 20 nm of siRNA against CCR5 or control siRNA against scrambled. After 5 h, cells were analyzed via intracellular flow cytometry for p‐Akt levels. (G) PKC412‐sensitive and PKC412‐resistant MOLM13 cell lines were serum‐starved for 12 h and incubated without or with 100 nm PKC412. After incubation, cells were analyzed via intracellular flow cytometric analysis for Bcl‐2. Significant differences are marked with (*): *P < 0.05, **P < 0.01, ***P < 0.001. Student’s t‐test. Error bars represent SD. *N* = 3 for (B–G).

CCR1, CCR3, CCR5, and GPR75 have been described as receptors for CCL5, with CCR5 being considered as the main receptor (Bruserud *et al.*, 2007; Ignatov *et al.*, 2006). All resistant MOLM‐13 cell lines expressed transcripts of the four receptors (Fig. 3A). Conversely, microarray data showed that CCR1 and CCR3 are decreased in the PKC412‐resistant cell lines (Fig. 4A). Therefore, RT–qPCR was conducted to examine whether the cell lines also differ in their expression levels of the main CCL5‐receptor CCR5. At the mRNA‐level, we detected a decrease of CCR5 mRNA in all resistant cell lines. Interestingly, the lowest level of CCR5 mRNA was detected in PKC F6 cells, which is the cell line with the highest CCL5 secretion (Fig. 3B). In flow cytometry, the level of CCR5 in the cell line with the highest CCL5 release, PKC F6, was significantly reduced (*P* < 0.001 for PKC F6; Fig. 3C). On the contrary, PKC412‐resistant cell lines with low CCL5 release showed a significantly increased number of CCR5‐positive cells in flow cytometry (*P* < 0.01 for PKC E9 and G7 and *P* < 0.05 for PKC F9).

It was shown that CXCR4 is colocalized with CCR5 in the same complex on the cell surface and that its expression is modulated similar to CCR5 by CCL5 (Wang *et al.*, 2004). In line with reduced CCR5 mRNA levels, microarray data implicated also downregulation of CXCR4 mRNA in the cell line PKC F6 (Fig. 4A). In flow cytometry, we detected a significantly lower surface expression of CXCR4 in PKC412‐resistant cell lines with high CCL5 release (*P* < 0.05 for PKC C5 and *P* < 0.01 for PKC F6), compared to sensitive cells (Fig. 3D). In contrast, the resistant cell lines with low CCL5 release showed a significant upregulation of CXCR4 compared to the sensitive MOLM‐13 cell line (*P* < 0.05 for PKC E9 and G7; *P* < 0.001 for PKC F9) (Fig. 3D). To exclude that this modulation is caused by the CXCR4‐ligand SDF‐1α, we measured SDF‐1α in the resistant cell lines and detected only low, subphysiological concentrations (Fig. 3E). CCL5 is a well‐known chemotaxis‐mediating factor in immune regulatory processes and is known to be released from AML cells in a subset of patients (Bruserud *et al.*, 2007; Reikvam *et al.*, 2019). Based on this fact, we hypothesized that aside from its PKC412‐resistance mediating effect, CCL5 may be a factor involved in interaction within the bone marrow niche and chemotactic processes between different clones of AML blasts *in vivo*. As expected, PKC412‐sensitive MOLM‐13 cells displayed significantly enhanced chemotaxis toward supernatant from PKC412‐resistant cell lines that show high CCL5 release (*P* < 0.01 for supernatant from PKC C5; *P* < 0.001 for supernatant from PKC F6; Fig. 3F). After having identified the reduction in cell surface receptors CCR5 and CXCR4 in PKC412‐resistant cell lines with high CCL5 release and the upregulation in cell lines with low CCL5 release, we examined whether PKC412 resistance can be abolished by blocking the CCR5‐CXCR4 receptor complex. For this purpose, we applied the CXCR4 antagonist plerixafor representatively in three cell lines (PKC E9, F6, F9). Indeed, we found significantly reduced levels of Annexin^‐^/7‐AAD^‐^ viable cells in the two cell lines PKC E9 and F9, which showed high values of CCR5/CXCR4‐positive cells, after coincubation with PKC412 and plerixafor (250 nm or 1 μM) for 36 h (*P* < 0.05) by flow cytometry (Fig. 3G,H).

### 3.4 PKC412‐resistant cell lines express mediators of survival and proliferation

To identify pathways that are involved in resistance against PKC412 induced by CCL5, we subjected RNA from three different PKC412‐resistant cell lines (PKC C5, E9, F6), as well as from sensitive MOLM‐13 cells to microarray analysis. Important signaling mediators for survival/proliferation pathways, Akt1, Akt3, Stat1, and Stat5, were significantly upregulated in the PKC412‐resistant and high CCL5 expressing cell lines PKC C5 and F6 compared to PKC412‐sensitive MOLM‐13 cells and the CCL5 low expressing PKC412‐resistant cell line PKC E9 (Fig. 4A). Based on the microarray results, we subjected PKC412‐sensitive MOLM‐13 cells and the five PKC412‐resistant cell lines to intracellular flow cytometry analysis of p‐Akt and p‐Stat5. Compared to PKC412‐sensitive cells, we found significantly increased numbers of cells positive for p‐Akt in PKC F6 and PKC F9 in the presence of PKC412 at 100 nm with the cell line PKC F6 showing the highest proportion of p‐Akt positive cells (*P* < 0.001 for PKC F6; *P* < 0.01 for PKC F9) (Fig. 4B). Flow cytometry for p‐Stat5 revealed significantly increased levels of p‐Stat5‐positive cells in the cell lines PKC C5 and E9 (*P* < 0.05 for PKC C5; *P* < 0.01 PKC E9) (Fig. 4C).

To examine if the increased numbers of p‐Akt positive cells in the cell line PKC F6 under PKC412 are induced by CCL5‐CCR5/CXCR4 signaling, we subjected this cell line to plerixafor (1 µm) for different time intervals. Shorter plerixafor treatment periods (1 or 2 h) led to significantly reduced p‐Akt positive cells (*P* < 0.001 for 1h and *P* < 0.01 for 2h) (Fig. 4D). Similarly, treatment with anti‐CCL5 for 1 h abrogated levels of elevated p‐Akt positive cells, but at high concentration of 2.5 µg/µL only (*P* < 0.001, respectively; Fig. 4E).

To analyze, if the CCL5‐CCR5 axis could be overcome by knockdown of CCR5, we transfected the PKC412‐resistant cell line PKC F6 with siRNA and analyzed p‐Akt by intracellular flow cytometry. This cell line showed the highest number of p‐Akt positive cells among the resistant cell lines. We found a reduction of p‐Akt positive cells after transfection (*P* < 0.05) (Fig. 4F).

To analyze whether increased p‐Akt may lead to upregulation of the antiapoptotic protein Bcl‐2, we performed intracellular flow cytometry for Bcl‐2 in the PKC412‐sensitive and the five PKC412‐resistant cell lines upon addition of PKC412. Compared to PKC412‐sensitive cells, we found a significant increase in the number of cells positive for intracellular Bcl‐2 in the PKC412‐resistant cell line PKC F6 at 100 nm PKC412 (*P* < 0.001; Fig. 4G). Additionally, a higher proportion of cells in the cell line PKC F9, which had shown slightly elevated levels of p‐Akt, was found to be positive for intracellular Bcl‐2 compared to PKC412‐sensitive MOLM‐13 cells at 100 nm PKC412 (*P* < 0.001) (Fig. 4G).

### 3.5 Overexpression of CCL5 in PKC412‐sensitive MOLM‐13 cells mediates enhanced resistance to PKC412

**Figure 5:**
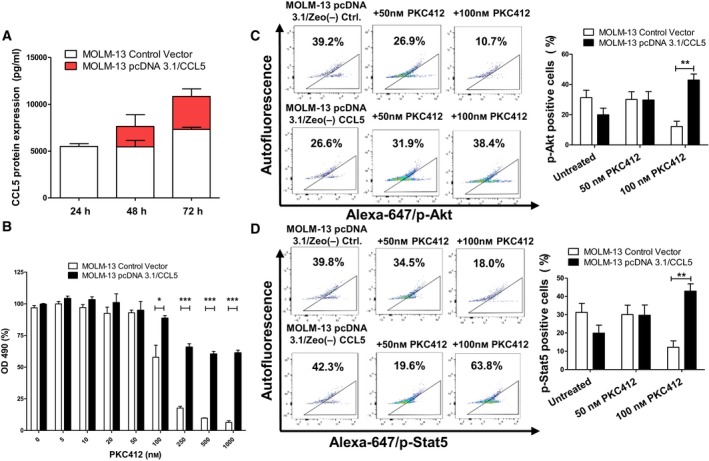
CCL5 overexpression in MOLM‐13 sensitive cells leads to sustained resistance to PKC412 (A) MOLM‐13‐sensitive cells were transiently transfected with a CCL5‐overexpressing vector or an empty control vector. Expression on protein level was higher 72 h after transfection. (B) TKI‐sensitive MOLM‐13 cells were transiently transfected with a CCL5 expressing vector or the corresponding empty control vector and were replated in serum‐free medium 48 h after transfection followed by treatment with PKC412 at the indicated concentrations. Proliferation was measured using MTS assay 48 h after addition of PKC412. (C) and (D) TKI‐sensitive MOLM‐13 cells were transiently transfected with a CCL5‐expressing vector or an empty control vector and were replated into serum‐free medium 48‐h after transfection followed by treatment with 50 and 100 nm PKC412. After 12 h, cells were analyzed via intracellular flow cytometric analysis for the levels of (C) p‐Akt and (D) p‐Stat5. Significant differences are marked with (*): *P < 0.05, **P < 0.01, ***P < 0.001. Student’s *t*‐test. Error bars represent SD. *N* = 3 for (A–D).

To verify whether CCL5 is sufficient to cause PKC412‐resistance, we transfected PKC412‐sensitive MOLM‐13 cells with a CCL5‐encoding vector (Fig. 5A). Forty‐eight hours after transfection, the cells were replated for MTS assay. Significant more cells were viable at 50 to 1000 nm PKC412 compared to control cells (*P* < 0.01) (Fig. 5B). In addition, CCL5 overexpressing cells showed a significantly higher number of cells positive for p‐Akt at 100 nm (*P* < 0.01) and p‐Stat5 at 100 nm PKC412 (*P* < 0.01) compared to cells transfected with a control vector (Fig. 5C,D).

### 3.6 Increased CCL5 expression after PKC412 treatment in primary human AML blasts

**Figure 6:**
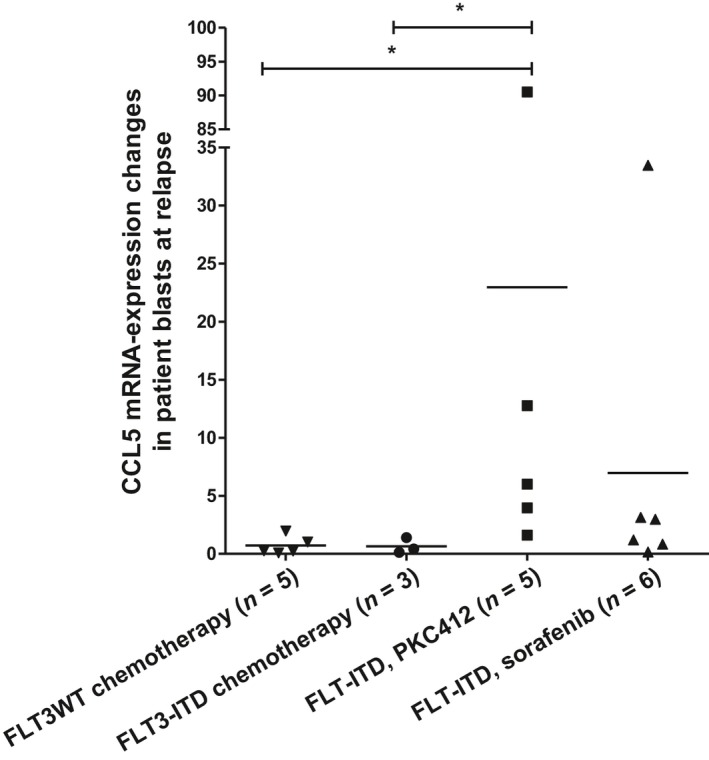
Induction of CCL5 expression in blast persistence or at relapse after anticancer treatment. Quantitative PCR for CCL5 with cDNA/RNA isolated from blasts (Ficoll isolation of mononuclear cells: percentage of blasts: 20–99%) of AML patients. Expression changes of CCL5 in blasts at blast persistence or relapse after chemotherapy (left), PKC412‐treatment (middle), or sorafenib treatment (right) are shown. Significant differences are marked with (*): *P < 0.05.

Finally, to examine the role of CCL5 in TKI‐resistant AML, we analyzed CCL5 expression by RT–qPCR in blast cells isolated from FLT3‐ITD‐mutated AML patients at baseline and at relapse or at the time of blast persistence. Patients were either treated by chemotherapy only (*n* = 3 for FLT3‐ITD‐positive patients and *n* = 5 for FLT3‐ITD WT patients), by PKC412 plus chemotherapy (*n* = 5), or by sorafenib plus chemotherapy (*n* = 6). We detected significantly elevated CCL5 expression in blasts that persisted or recurred after PKC412 treatment compared to blasts from patients treated with sorafenib and/or chemotherapy, with values up to 90‐times higher as compared to baseline (Fig6).

## 4 Discussion

Patients with FLT3‐mutated AML commonly develop resistance under treatment with target‐specific drugs as PKC412. In only a minority of cases can resistance to FLT3‐TKIs be tracked to secondary resistance mutations in the FLT3 gene (von Bubnoff *et al.*, 2010; Heidel *et al.*, 2006; Smith *et al.*, 2012). Our data demonstrate that the chemokine CCL5 is involved in the emergence of drug resistance against PKC412. This hypothesis is supported by a) the high release of CCL5 from some of our FLT3‐ITD inhibitor resistant MOLM‐13 AML cell lines in which underlying secondary resistance mutations in the FLT3‐gene were excluded, b) significantly higher viability of PKC412‐sensitive MOLM‐13 cells in the presence of PKC412 after both short‐ and long‐term CCL5 treatment, as well as the abrogation of this effect by the addition of the CCR5‐inhibitor TAK‐779, c) increased survival of PKC412‐sensitive MOLM13 cells upon PKC412 treatment when transfected with a CCL5 overexpressing vector, d) inverse correlation of the CCL5 receptor CCR5 and CXCR4 expression levels and released CCL5 levels in the PKC412‐resistant cell lines, and e) attenuated survival of CXCR4 inhibitor plerixafor‐treated cells. Further evidence for our hypothesis is given by the fact that we also detected a significantly higher expression of CCL5 with values increased up to 95‐times of controls. Our data are in agreement with previous studies where CCL5 was linked to chemotherapy and TKI resistance. Thus, CCL5 was identified as an associated mediator of drug resistance to tamoxifen, saracatinib, and chemotherapeutics in breast cancer and several chemotherapeutics like doxorubicin, cisplatin, or taxanes were shown to upregulate the CCL5‐CCR1/CCR5 pathway in human melanoma cell lines or prostate cancer cells (Fang *et al.*, 2018; Ullah *et al.*, 2019; Yi *et al.*, 2013). For the latter entity, production of CCL5 from tumor‐infiltrating CD4^+^ lymphocytes has just recently been shown (Xiang *et al.*, 2019). Furthermore, it was shown that pretreatment with CCL5 and other cytokines protected lung cancer cells against doxorubicin (Levina *et al.*, 2008). With regard to AML, Yang *et al. *(2014) demonstrated that cytokines released by stromal cells mediate resistance to FLT3‐TKIs such as quizartinib, although the involved cytokines were not identified (Yang *et al.*, 2014). Therefore, CCL5‐mediated tumor cell survival after drug treatment seems to be a common mechanism in cancer. However, to our knowledge, our results are the first that support the hypothesis that in blood cancer/AML, increased CCL5 release from AML blasts mediates resistance to PKC412.

To understand the mechanism of action for CCL5 mediated drug resistance, we explored CCL5‐receptor expression. We detected significantly lower levels of CCR5‐positive cells in cell lines with high CCL5 release and vice versa. Downregulation of CCR5 by CCL5 was shown to occur via cytoplasmic internalization into vesicles (Amara *et al.*, 1997). Hence, downregulation of CCR5 in resistant MOLM‐13 cell lines does not preclude a long‐term protective effect of CCL5 against PKC412 in the drug‐sensitive MOLM‐13 cells. CXCR4 is expressed on solid and hematologic cancers including AML and has been demonstrated to be present in a complex with CCR5 on the cell surface (Contento *et al.*, 2008; Singer *et al.*, 2001; Wang *et al.*, 2004). In AML, this receptor plays an important role for the aberrant homing of leukemia cells (Sison *et al.*, 2013; Weitzenfeld and Ben‐Baruch 2014). Analogous to CCR5 expression, we observed that the cell line with the highest release of CCL5 displayed the lowest levels of CXCR4‐positive cells, reflecting receptor downregulation in response to CCL5. The concept of receptor downregulation as a response to high amounts of CCL5 is supported by the observation that CCR5 and CXCR4 are colocalized on the cell surface and thus are regulated in the same manner upon ligand binding (Wang *et al.*, 2004).

Notably, not all PKC412‐resistant cells displayed elevated CCL5 expression. As shown by previous studies, various CCL5‐independent mechanisms might induce PKC412 resistance. Thereby, secondary FLT3‐mutations are most frequently observed under clinical circumstances to mediate resistance against FLT‐ITD TKIs (Daver *et al.*, 2015; Williams *et al.*, 2013). In our approach, we first excluded secondary mutations in the FLT3‐gene by Sanger sequencing. In general, mutations of the FLT3 receptor or, more often, upregulation of downstream signaling pathways independently of FLT3 are considered to underlie TKI resistance (Ghiaur and Levis 2017). Furthermore, metabolic changes with upregulation of glycolysis and downregulation of mitochondrial respiratory chain activity (Huang *et al.*, 2016), higher levels of constitutively phosphorylated Axl (Park *et al.*, 2015), increased pim kinases (Green *et al.*, 2015), or upregulation of surface FLT3‐ITD (Weisberg *et al.*, 2011) were all described for FLT3‐ITD inhibitor resistance. As we detected elevated levels of the cytokine CCL5 only in the supernatant of two PKC412‐resistant cell lines and multiple studies reported CCL5 to mediate chemotherapy and TKI resistance, we focused on this cytokine and its receptor. We also provide evidence that the entire CCL5/CCR5 axis is involved in PKC412 resistance in MOLM13 cells: PKC412‐resistant cell lines that did not show elevated CCL5 levels displayed higher CCR5 expression (Figs 1 and 3). Thus, we found that we can group the PKC412‐resistant cell lines into two groups with either CCL5 overexpression and CCR5 downregulation or vice versa. We conclude that in cases of low CCL5 secretion upregulation of the CCR5 receptor might lead to increased CCL5/CCR5 signaling eventually mediating resistance to PKC412 via increased downstream signaling pathways including p‐Stat5 or p‐Akt.

At the molecular level, sustained survival might be caused by upregulation of proliferation promoting pathways and antiapoptotic proteins. In RNA microarrays, PKC412‐resistant cell lines that secreted high levels of CCL5, overexpressed Stat1, Akt1, Akt3, and p‐Stat5. It has previously been shown that the PI3K/Akt pathway is involved in resistance to the FLT3‐inhibitor sorafenib (Lindblad *et al.*, 2016). Indeed, we were able to verify the presence of a significantly elevated number of p‐Akt positive cells in the PKC412‐resistant cell lines PKC F6 and F9. Notably, the cell line PKC F6, which secreted high levels of CCL5, showed the highest proportion of p‐Akt‐positive cells. Additionally, the levels of p‐Stat5‐positive cells in the cell lines PKC E9 and PKC C5 were increased, implicating that the CCL5‐CCR5/CXCR4 axis activates the PI3K/Akt or Jak/Stat pathway. Moreover, treatment with plerixafor, which is an inhibitor to CXCR4, led to a reduced number of p‐Akt‐positive cells in the cell line PKC F6. Further evidence for the role of p‐Akt as a key mediator for CCL5‐induced PKC412‐resistance is given by the fact that transfection of PKC412‐sensitive MOLM‐13 cells with a CCL5‐encoding vector led to an increased number of p‐Akt‐positive cells under PKC412 treatment. Additionally, treatment with siRNA targeting the CCL5‐receptor CCR5 led to a reduced proportion of p‐Akt‐positive cells. Moreover, in the same PKC412‐resistant cell lines, a higher amount of cells stained positive for intracellular antiapoptotic protein Bcl‐2, presumably because p‐Akt regulates activation of the downstream target Bcl‐2 as described (Zhang *et al.*, 2011). Therefore, the PI3K/Akt pathway will be of particular interest in the future.

It was reported that CCL5 is secreted by blasts from AML patients (Bruserud *et al.*, 2007). However, apart from mediating chemotaxis of immune cells, the function of CCL5 released by AML blasts remained unclear. Our study provides evidence that the CCL5 level might predict clinical relapse in AML after TKI treatment. Interestingly, in blasts from patients that did not achieve complete hematological remission or relapsed after sorafenib treatment, we detected a slight increase in CCL5 expression, whereas in blasts from PKC412‐treated patients, the CCL5 expression was greatly increased at the time of relapse. These results indicate that CCL5 plays a role in TKI resistance, especially for PKC412. This is supported by a previous study that demonstrated that cytokines released from stromal cells could protect human AML blasts from FLT3‐TKI‐induced apoptosis (Yang *et al.*, 2014). However, due to the small patient number, our data do not allow definitive conclusions to be made. A study with a larger cohort and analysis of multiple cytokines at once will be necessary to examine the impact of CCL5 alone or in combination with other cytokines as a biomarker for PKC412 resistance in AML.

Mechanistically, it has been shown that CCL5 released by human AML cells affects the migration of immune cells (Olsnes *et al.*, 2006). It has been shown that human AML blasts release a large number of different cytokines, many of these present in clusters (Bruserud *et al.*, 2007). Furthermore, we investigated the chemotactic behavior of PKC412‐sensitive MOLM‐13 cells toward supernatants of PKC412‐resistant MOLM‐13 cells and found a higher chemotactic level toward supernatants from resistant cell lines with high CCL5 release compared to supernatants obtained from PKC412‐resistant cell lines with low CCL5 release. CCL5 was shown to play an important role in the chemotaxis and homing of AML cells (Weitzenfeld and Ben‐Baruch 2014). Therefore, secretion of CCL5 by AML blasts might not only be interesting for drug resistance but also for an altered chemotactic behavior of AML cells.

## 5 Conclusions

Our results show that CCL5 is released *in vitro* by PKC412‐resistant AML cell lines and *ex vivo* by AML blasts from FLT3‐ITD‐mutated and PKC412‐treated patients at relapse. CCL5 acts as a mediator of treatment resistance and aberrant cell migration. It might play a role as a biomarker to monitor therapy response to PKC412, which was recently approved for the treatment of FLT3‐mutated AML, and might be of potential interest as a treatment target for future therapies overcoming drug resistance. However, further studies are necessary to confirm the predictive value of CCL5 for the development of resistance to FLT3 inhibitors in FLT3‐mutated AML and to delineate the significance of receptor binding and its precise effect on downstream signaling. Moreover, it remains to be determined if other cytokines participate together with CCL5 *in vivo* in the regulation of FLT3‐TKI resistance.

## Conflict of interest

NvB received research funding from Novartis. The other authors have no conflict of interest to declare.

## Author contributions

SW planned, performed, and analyzed the experiments and wrote the manuscript. MR planned and performed some of the experiments and wrote the manuscript. PK provided the resistant cell lines and assisted with the cytokine array. MF and DH assisted with the FACS experiments. CE assisted with the experiments. AC helped with the vector and siRNA design. GA and MB conducted all processes for microarray analysis. US planned and helped to establish the ELISA and helped with the analysis of some of the experiments (ELISA). JD revised the manuscript; and NvB designed the research, planned the experiments, analyzed the data, and wrote the manuscript. All authors read and approved the final manuscript.

## Supporting information


**Fig. S1.** PKC412‐resistant cell lines show increased levels of viable cells after 36 h of treatment with PKC412 in flow cytometry PKC412‐resistant cell lines were cultivated at 100 nm PKC412. For flow cytometric analysis of apoptosis, they were exposed subsequently for 36 h at 50 or 100 nm PKC412. In addition, PKC412 was withdrawn from every cell line for 24 or 72 h, respectively, followed by treatment with 50 or 100 nm PKC412 for 36 h. The PKC412‐sensitive MOLM‐13 cell line as control is depicted for each approach (separated by dotted line)

## Data Availability

The datasets generated during and/or analyzed during the current study are available from the corresponding author on reasonable request.
