# Supplementary figures and images for: CCL5 mediates target‐kinase independent resistance to FLT3 inhibitors in FLT3‐ITD‐positive AML
Source: Mol Oncol. 2020 Feb 13;14(4):779–94. doi: 10.1002/1878-0261.12640 (PMC7138400; doi:10.1002/1878-0261.12640)

# Suppl. Figure 1

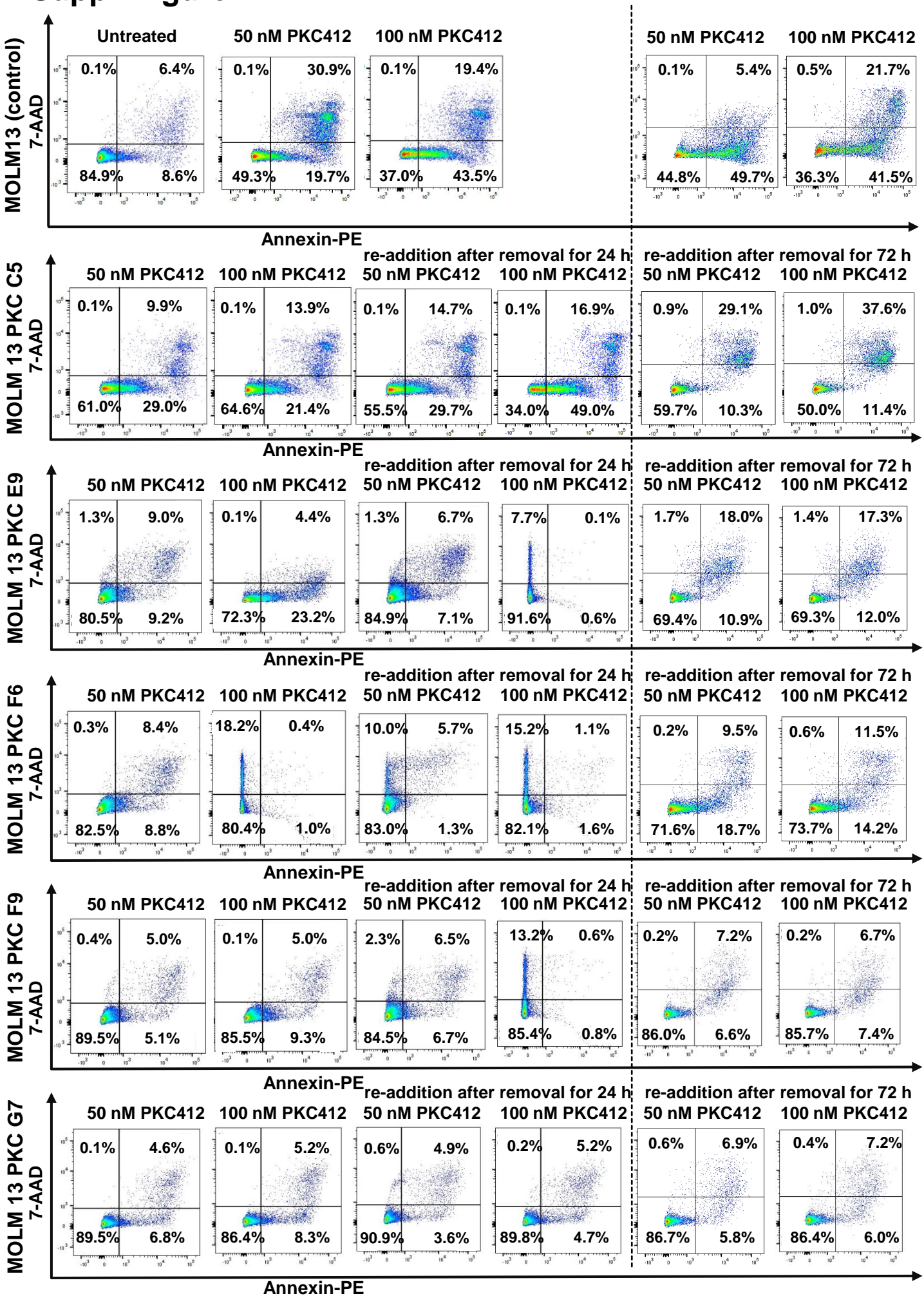

Supplement: Supplementary file 1 — Fig. S1. PKC412‐resistant cell lines show increased levels of viable cells after 36 h of treatment with PKC412 in flow cytometry PKC412‐resistant cell lines were cultivated at 100 nm PKC412. For flow cytometric analysis of apoptosis, they were exposed subsequently for 36 h at 50 or 100 nm PKC412. In addition, PKC412 was withdrawn from every cell line for 24 or 72 h, respectively, followed by treatment with 50 or 100 nm PKC412 for 36 h. The PKC412‐sensitive MOLM‐13 cell line as control is depicted for each approach (separated by dotted line) [file MOL2-14-779-s001.pdf]
